# Retraction: Novel electrospun polyurethane grafts for vascular access in rats

**DOI:** 10.1177/11297298251316657

**Published:** 2025-02-06

At the request of the Journal Editor and Sage the following article has been retracted:

Alqahtani SS, Sloff M, Sawo P. Novel electrospun polyurethane grafts for vascular access in rats. The *Journal of Vascular Access*. 2022;0(0). doi:10.1177/11297298221131393

Sage was contacted by the former supervisor of one of the authors of this article, noting that the authors were not authorized by the institution to use data for the article and that the article was published without the co-authors’ consent.

The co-authors responded to Sage’s queries and confirmed that they did not provide consent. The corresponding author notes that all authors provided consent.

Due to unresolved concerns around the authorship of the article, the Journal Editor retracts this article.

The corresponding author did not agree with the retraction.

